# The effect of mixing and consistency on cellulose cationization

**DOI:** 10.1016/j.heliyon.2019.e01349

**Published:** 2019-03-18

**Authors:** Pia Willberg-Keyriläinen, Pauliina Pitkänen, Janne Hulkko, Martta Asikainen, Harri Setälä

**Affiliations:** VTT Technical Research Centre of Finland Ltd, Tietotie 4E, P.O Box 1000, FI-02044 VTT, Finland

**Keywords:** Organic chemistry, Natural product chemistry

## Abstract

Traditional chemical modifications of cellulose are usually done at low or medium consistencies. Processing at high solids content is expected to provide a leap forward in reduction of manufacturing costs such as minimization of chemical use, energy consumption and decreasing processing equipment size, while at the same time increasing reaction efficiency. In this research, high consistency modifications of cellulose were studied through cationization reaction. Four different laboratory scale high consistency reactors were tested and the effect of mixing on fiber properties was analyzed. All reactors decreased cellulose fiber length and no significant difference between cellulose starting consistencies and mixing time on fiber properties were found. The cationization reaction efficiency increased as the cellulose starting consistency increased in all of the tested reactors. In addition, mercerization of pulp as pretreatment, significantly increased reaction efficiency.

## 1 Introduction

Synthetic polymeric quaternary ammonium compounds are a class of conventional polyelectrolytes [1]. These materials are used widely in many applications such as adsorbent materials for dyes or heavy metal ions, flocculants in wastewater treatment and in cosmetics. Their disadvantages are that they are petroleum based, non-biodegradable, and in many cases toxic to aquatic organisms [2]. Cationized cellulose as water-soluble derivatives or fibers are one important group of industrially produced cellulose derivatives that can be used to replace synthetic polyelectrolytes. Their advantages are low cost, biodegradability and low toxicity [3, 4]. Cationized celluloses and other polysaccharides have been studied intensively in recent decades as flocculants, paper additives, hair care products and absorbent materials [3, 4, 5, 6, 7, 8].

Most cationic celluloses are ether derivatives prepared usually using glycidyltrimethylammonium chloride (GTAC) or 3-chloro-2-hydroxypropyltrimethylammonium chloride (CHPTAC) and catalyzed by a base such as sodium hydroxide (NaOH). The reaction efficiency (RE) of cationization using either GTAC or CHPTAC has been studied intensively in the cationization of starches [9, 10, 11]. The reaction efficiency is dependent on several reaction parameters: such as the type of reagents, molar ratios of reagents (cationization reagent/catalyst/cellulose/water), reaction time and temperature. Earlier reports indicated that the amount of needed NaOH was much lower and also RE higher when GTAC was used as the cationization reagent instead of CHPTAC [12, 13]. A clearly improved reaction efficiency was also observed when the molar ratio of GTAC/AGU was between 0.5–1.0. The reaction temperature and the molar ratio of NaOH/AGU are also important reaction parameters and should be optimized [9, 11, 12, 13].

Often mercerization treatment with strong NaOH solutions is used to improve the reactivity of cellulose and reaction efficiency before the final cationization step. This alkali treatment has been observed to remarkably increase reaction efficiency depending on treatment time and alkalinity. The alkaline pretreatment produces changes in intramolecular and intermolecular bonds of cellulosic fibers. It changes cellulose crystalline forms (allomorphs) from I (Iα/Iβ) to cellulose II, and at the same time it can increase the amount of amorphous cellulose [14]. Simultaneous mechanical treatment and mercerization has also been observed to increase the reaction efficiency of cationization [15].

In this study, we have studied the effect of mixing on fiber properties when high consistency reactors were used. We have used the GTAC cationization method as a model reaction to compare the effect of reaction parameters such as cellulose consistency, reaction time, and amount of NaOH, and especially the effect of reactor type in high consistency reactions. The cellulose consistency varied from 10 wt% up to 70 wt % using two different pulps. Also, the effect of cellulose mercerization on reaction efficiency was studied.

## 2 Materials and method

### 2.1 Materials

The cellulose materials used in this research were never-dried Enocell hardwood dissolving pulp (HWD) produced by Stora Enso, Finland and never-dried softwood kraft-grade (SWK) pulp produced by MetsäFibre, Finland. Glycidyltrimethylammonium chloride (GTAC, 70% solution in water) was purchased from Chemigate, Finland. All other commercial reagents were purchased from Sigma-Aldrich in the highest purity grade and were used as received.

### 2.2 Chemical composition of the pulp

The pulps were air dried and ground using a Wiley mill. To determine carbohydrate composition, the samples were hydrolyzed with sulfuric acid and the resulting monosaccharides were determined by HPAEC with pulse amperometric detection (Dionex ICS 3000A equipped with CarboPac PA1 column) according to the NREL method [16, 17]. Carbohydrates were calculated in their anhydroform with correction factors of 0.9 for hexoses and 0.88 for pentoses.

### 2.3 Size exclusion chromatography (SEC)

The relative molar masses of the pulp samples were determined by size exclusion chromatography (SEC) measurements in 0.8% LiCl/DMAc eluent (0.36 ml/min, 80 °C) using MiniMix columns equipped with a Waters 2414 Refractive Index Detector (Waters, Milford, USA). The relative molar mass distributions and average molar masses (Mn, Mw) were calculated against Pullulan Standards (6 100–1 600 000 g/mol). For SEC measurements, the pulp samples were dissolved in an 8% LiCl/DMAc, according to the solvent exchange method, with ethyl isocyanate derivatization to enhance the dissolution [18].

### 2.4 Mechanical pretreatment tests

Different high consistency reactors were used for mechanical pretreatment tests: DIT reactor (Design Integrated Technology, USA), Lödige reactor (Gebrüder Lödige Maschinenbau GmbH, Germany), LIST reactor (LIST Technology AG, Switzerland) and IKA reactor (IKA Labortechnik, Germany). Detailed model information is tabulated in Table 2. In mechanical pretreatment tests, the pulp was mixed 1 and 5 h at 50 °C and the mixing speed was 40–250 rpm, depending on the reactor.

### 2.5 Fiber analysis

Fiber properties (length-weighted average fiber length, kinks and fines content) after mechanical pretreatment tests were analyzed using the FiberMaster instrument (Fibermaster STFI, Lorenzen&Wettre, Sweden).

### 2.6 Optical microscopy analysis

Optical microscopy studies were done with a Nikon Eclipse Ci Light Microscope (Nikon, UK) using x4 magnification.

### 2.7 Cellulose mercerization

The mercerization was performed in a 5 L glass reactor using the slightly modified method published by Mansikkamäki et al. [19, 20]. Never-dried hardwood dissolving pulp (HWD) was mixed with deionized water and isopropanol. Then 50 wt% sodium hydroxide solution (2.3 eq/AGU) was added slowly. The mixture was stirred overnight at room temperature. Then pH was adjusted to 7–8 with concentrated sulfuric acid. Pulp was filtrated and washed several times with water, and finally with aqueous 50 wt% isopropanol.

### 2.8 Cellulose cationization

Cationization of cellulose was conducted in a heterogeneous method using different high consistency reactors. The cellulose, sodium hydroxide water solution (10%, 0.16 equivalent per cellulose anhydroglucose unit (AGU) and glycidyltrimethylammonium chloride (GTAC, 1eq/AGU) were mixed together and the mixture was stirred 6–24 h at 45 °C. Purification was performed by washing the mixture with ethanol and dialyzing against water using a membrane (Biotech RC) with a cut-off of 3.5–5 kD.

### 2.9 Nitrogen content and reaction efficiency

The degree of substitution (DS) of the cationic cellulose derivatives was determined based on the nitrogen content (N%) of the samples by the Kjehldahl titration method according to the method published by Bendoraitiene et al. [10]. The reaction efficiency (RE) indicates the percentage of added cationic reagent that has reacted with cellulose. RE was calculated by dividing DS by amount of added GTAC (mol/AGU) and multiplied by 100%.

## 3 Results and discussion

### 3.1 Monosaccharide composition of pulps

Two different cellulose pulps were used in this research: hardwood dissolving pulp (HWD) and softwood kraft-grade pulp (SWK). The molecular masses of the starting pulps were 390 kDa and 860 kDa for HWD pulp and for SWK pulp, respectively. Carbohydrate composition was determined for pulps and the results are shown in Table 1. According to the carbohydrate composition results, glucose is the main monosaccharide in all samples and its primary source is cellulose. Cellulose content of HWD was 79.8% and it was somewhat higher than cellulose content of SWK (73.4%). The xylan is the major hemicellulose in both pulps and its contents are 4.7% and 8.0% for HWD and SWK, respectively. In addition, the SWK pulp also contains 5.4% glucomannan.

**Table 1:** Carbohydrate composition of the HWD and SWK pulps, expressed as mg monosaccharide/100mg dry pulp.

Composition	HWD	SWK
Glucose	88.7	81.6
Xylose	5.4	8.3
Mannose	0.3	6.0
Arabinose	<0.1	0.8
Galactose	<0.1	0.2
Fructose	<0.1	<0.1
Rhamnose	<0.1	<0.1
Monosaccharides total	95	97
Polysaccharides∗	85	87

∗ Anhydrous correction factor 0.9 for glucose to cellulose and 0.88 for xylose to xylan.

**Table 2:** Detailed information about high consistency reactors and parameters.

Reactor	Model	Maximum volume (L)	Load (g of dry cellulose)	Mixing speed (rpm)	Mixing mechanism
DIT	2CV	0.37	20	400	vertical dual helical-conical blades
IKA	HKD-T0.6	0.6	15	36	horizontal dual z-shaped blades
LIST	9476	3	200	100	horizontal dual multi-anchor blades
Lödige	M5R	5	200	300	horizontal one blade

### 3.2 Mechanical pretreatment tests

**Fig. 1:**
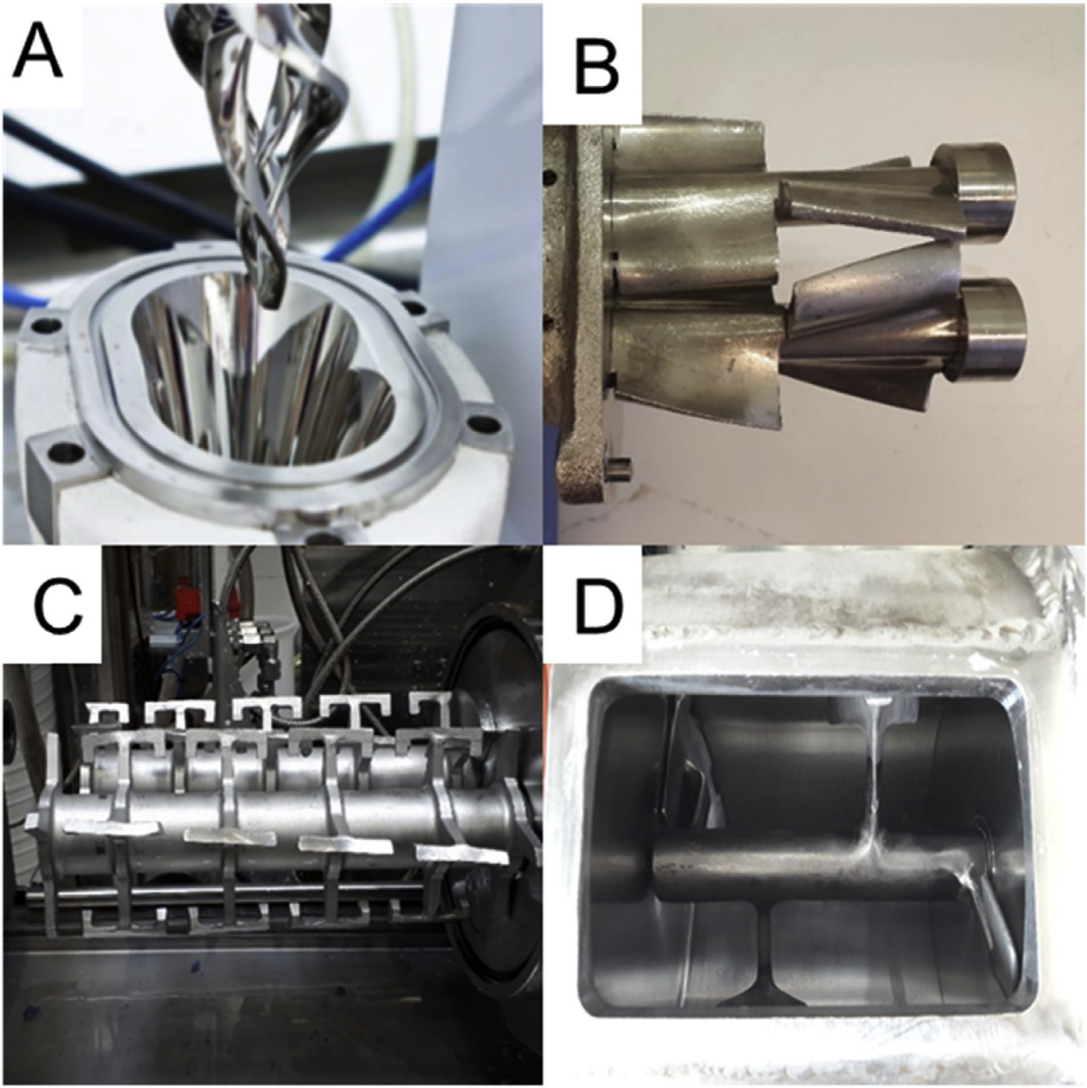
Mixing mechanism of used high consistency reactors A) DIT reactor, B) IKA reactor, C) LIST reactor and D) Lödige reactor.

In this research, four different high consistency reactors were tested and detailed information about the used high consistency reactors is listed in Table 2 and mixing mechanism of high consistency reactors are shown in Fig. 1.

The effect of high consistency reactors on cellulose fibers was studied using a FiberMaster analyzer and molar masses were analyzed using SEC measurements. For these analyses, the hardwood dissolving pulp was mixed in reactors using a cellulose consistency of 10–50 wt%, and samples were taken after one and 5 h of mixing.

**Fig. 2:**
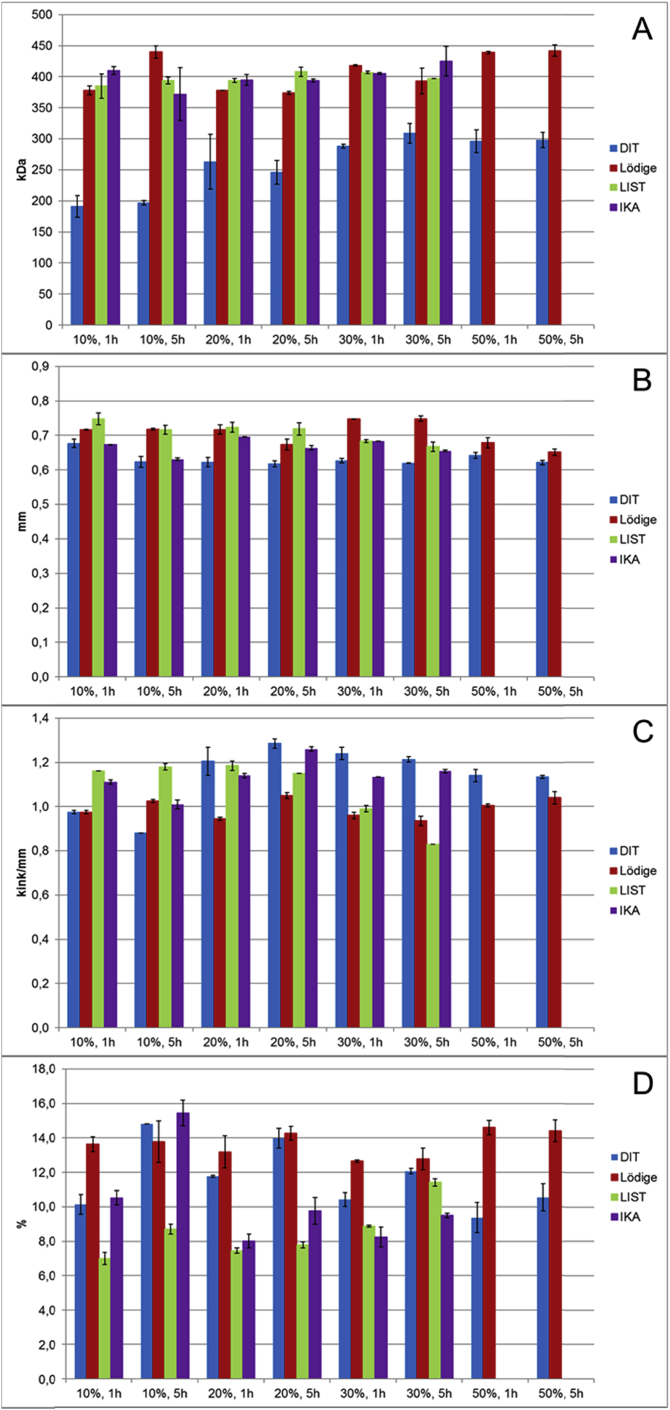
Effect of different high consistency reactors on HWD pulp properties and fiber dimensions A) molar mass, B) fiber length, C) amount of kinks and D) amount of fines.

The molar mass results (Fig. 2A) showed that the DIT reactor is the only reactor having an effect on cellulose molar mass. Using the DIT reactor, the molar mass decreased from 390 kDa to less than 200 kDa regardless of cellulose consistency. The mixing time had no effect on molar mass. The highest effect was observed with 10% consistency. This might be due to the shearing effect of the DIT reactor mixing system on cellulose fibers. The DIT reactor was the only tested reactor with a vertical mixing mechanism.

All reactors decreased cellulose fiber length and no significant difference between cellulose starting consistencies and mixing time were found (Fig. 2B). The fiber length was decreased about 25% during mixing in the DIT reactor, whereas reduction was 5–16% for other reactors.

Earlier studies reported that shearing at high consistency causes kinks in fibers [21]. The Lödige reactor was the only tested reactor not increasing the amount of kinks/mm (Fig. 2C). This indicates that the mixing mechanism of the Lödige reactor is the most delicate and there is no shear generated in the reactor. When the LIST reactor was used, the amount of kinks increased when cellulose consistency decreased. This means that the drier the material, the less resistance to the stirring mechanism. When the DIT and IKA reactors were used, the opposite phenomenon was observed and the amount of kinks was increased, when cellulose consistency increased.

The amount of fines (<0.2 mm) increased significantly from 7% to 14% when DIT and Lödige reactors were used (Fig. 2D), while with LIST and IKA reactors the amount of fines increased only slightly. The longer mixing time increased the fines amount, whereas cellulose consistency has only a small effect, with the exception of 10% cellulose in the IKA reactor, in which the amount of fines increased abnormally.

Before the actual cationization experiments, the stirring effectiveness and drifting rate of chemicals were also studied by stirring the softwood kraft pulp (SWK) sample with dye (Nitor all-in-one marine blue textile dye). Cellulose dry content was 30% and the amount of dye corresponds to the total amount of liquids in the cationization reaction. According to the optical microscopy analysis, IKA and LIST reactors were the most effective reactors for mixing. After the 1 min of mixing, pulp was evenly colored, whereas it took 10 min for DIT and 10–30 min for Lödige reactors to reach an evenly distributed color. Although the pulp surface was evenly colored, penetration of color into fibers took 5–10 min longer.

### 3.3 Cellulose cationization

Cellulose cationization reactions were carried out using heterogeneous reaction method. The target was to modify only the cellulose surface and thus only 1 equivalent of glycidyltrimethylammonium chloride (GTAC) was used as a reagent. It has been earlier reported [22] that lower amount of reagent per AGU favors the etherification reaction over the hydrolysis reaction and increases the cationization reaction efficiency compared to using larger reagent quantities.

In the beginning, the reaction conditions were optimized by testing two different reaction temperatures, 45 °C and 75 °C, in the DIT reactor. Reaction efficiencies (RE) for 30 wt% HWD pulp were 17.5% and 16% in temperatures 45 °C and 75 °C, respectively. In the same temperatures, reaction efficiencies were 17% and 14% for 20 wt% HWD pulp. Bendoraitiene et al. [10] studied the effect of reaction temperature on the cationization reaction and found the same effect of RE decreasing when higher reaction temperatures are used. The reason is that side reactions of GTAC proceed faster at higher temperature, and thus negatively affect the desired cationization reaction efficiency. Therefore, a reaction temperature of 45 °C was chosen for further experiments. We also found that the reaction efficiency increased linearly for first 6 h of reaction time and thereafter it remained almost constant. This observation is in line with earlier reported by Prado et al. [23].

**Fig. 3:**
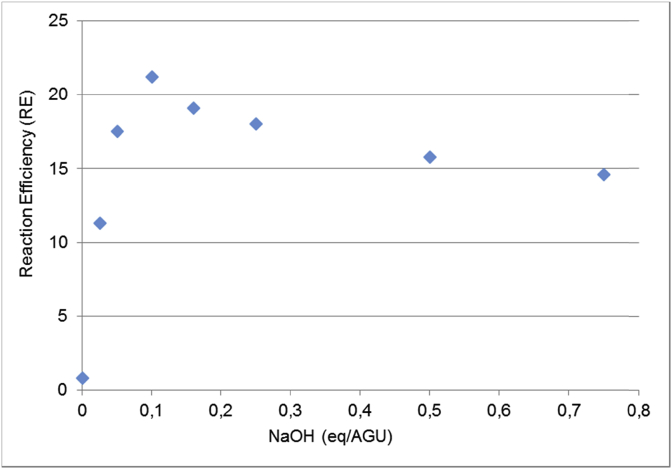
The effect of NaOH amount on reaction efficiency. 30% cellulose consistency and the DIT reactor were used.

The optimal NaOH amount for reaction efficiency was also optimized. The RE reached a maximum value when 0.16 eq/AGU of NaOH was used (Fig. 3). When the amount of NaOH was higher in the reaction mixture, the RE decreased. This is because the excess of NaOH favors epoxide degradation or side reactions [3, 11].

### 3.4 Effect of reactor on reaction efficiency

**Fig. 4:**
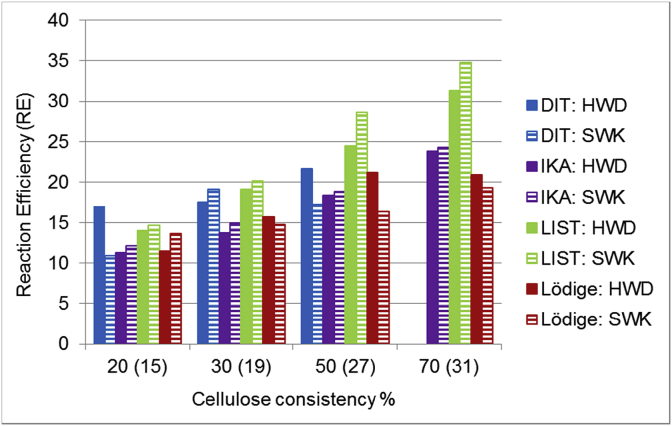
The effect of cellulose consistency (starting consistency without parentheses, end consistency with parentheses), cellulose pulp type and reactor type on reaction efficiency (Reaction time 24h, except LIST 6h).

The reaction efficiency (RE) increased as the cellulose starting consistency increased in the case of all tested reactors (Fig. 4). The change was the greatest when the LIST reactor was used, although the reaction time was only 6 h in the LIST reactor, while using DIT, Lödige and IKA reactors, the reaction time was 24 h. Also, the highest RE values were achieved when the LIST reactor was used. In that case, the RE increased from 15% to 35%, when softwood kraft pulp starting consistency increased from 20% to 50%.

When the DIT reactor was used, also quite high REs were achieved. However, when cellulose starting consistency was more than 50%, cellulose could no longer be mixed in the DIT reactor. These results indicate that the LIST and DIT reactors have the best mixing configuration for this reaction. It seems that the high shearing effect may decrease the crystallinity of cellulose, and thus enables better and more-even penetration of chemicals into the cellulose fiber structure.

Earlier it has been reported [10, 24, 25] that water reduces the RE of the cationization reaction due to side reactions of GTAC with NaOH. Thus, the water content has very big effect on the cationic reaction. Bendoraitiene et al. [10] also reported that the cationization reaction with GTAC proceeds only when a specific, small amount of water is present. When the water amount is doubled, the RE of cationization decreases. Zaman et al. [22] also observed that high water content can cause the hydrolysis of cationically modified cellulose and thus decrease RE. In their study, the RE increased threefold when cellulose starting consistency was increased from 20% to 70%. These observations are in line with our results, which state that the higher the cellulose consistency, the higher the reaction efficiency.

In most experiments the reaction efficiencies were better with SWK than with HWD. SWK pulp contains more hemicelluloses, which can react more easily under the used reaction conditions than cellulose and therefore might lead to a higher reaction efficiency.

### 3.5 Effect of pulp mercerization on reaction efficiency

Reactivity is an important quality parameter when chemical modification is considered. Reactivity is affected by chemical, structural and morphological properties of cellulose fiber [26].

Mercerization of cellulose is known to enhance reactivity where conversion of cellulose I to cellulose II took place [19]. The mercerization of cellulose in alcohol/water solutions is widely used in several industrial processes before chemical modifications such as carboxymethylation or hydroxypropylation. It has been shown that mercerization in aqueous alkaline alcohol mixtures is much faster than in pure aqueous alkaline systems [20].

**Fig. 5:**
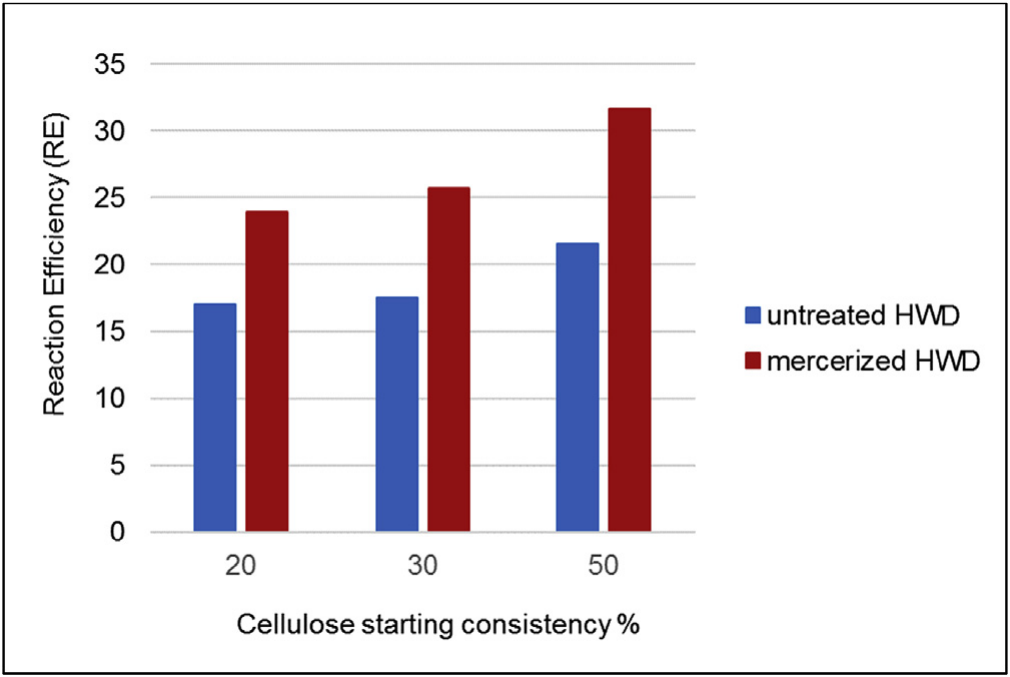
The effect of mercerization on reaction efficiency of cationized hardwood dissolving pulp when the DIT reactor was used.

In this study, we have also examined the effect of pulp mercerization on RE using an aqueous alkaline isopropanol mixture. Mercerized hardwood dissolving pulps were cationized in the DIT reactor and the results were compared to the untreated pulp (Fig. 5). Mercerization increased reaction efficiency significantly (40–50%) and the efficiency seemed to increase when cellulose starting consistency increased. De la Motte et al. [27] compared reaction efficiency of the cationization reaction with softwood kraft pulp, dissolving pulp and mercerized cellulose. They found that reaction efficiency doubled when mercerized pulp was used instead kraft pulp and dissolving pulp. Moral et al. [15] also concluded that mercerization decreased cellulose crystallinity and caused higher RE than without mercerization. This is because the amorphous part of cellulose is more reactive compared to the crystalline one. The mercerized cellulose also has a more open structure and the fibers possess a higher specific surface area and therefore the hydroxyl groups in cellulose are easier to access [28].

## 4 Conclusions

Four different high consistency reactors were tested and the effect of reactors on cellulose fibers was studied. All reactors decreased cellulose fiber length and no significant difference between cellulose starting consistencies and mixing time on fiber properties were found. The DIT reactor was the only reactor, which had an effect on cellulose molar mass and the Lödige reactor was the only tested reactor that did not increase the amount of kinks. The longer mixing time increased the amount of the fines fraction, whereas cellulose consistency had only a small effect on these parameters.

Cellulose cationization reactions were carried out using a heterogeneous reaction method. The RE increased as the cellulose starting consistency increased in the case of all tested reactors. The highest RE values were achieved when the LIST reactor was used, because the high strain effect enabled better and more-even penetration of chemicals into the cellulose fiber structure. The effect of mercerization on reaction efficiency was also tested. Mercerization as a pulp pretreatment increased RE significantly and the effect was increased when cellulose starting consistency was increased.

## Declarations

### Author contribution statement

Pia Willberg-Keyriläinen, Pauliina Pitkänen: Conceived and designed the experiments; Performed the experiments; Analyzed and interpreted the data; Contributed reagents, materials, analysis tools or data; Wrote the paper.

Martta Asikainen: Conceived and designed the experiments; Performed the experiments; Wrote the paper.

Janne Hulkko, Harri Setälä: Conceived and designed the experiments; Analyzed and interpreted the data; Wrote the paper.

### Funding statement

This work was part of the New Fiber Products project of the CLIC Innovation Ltd. This work was also supported by Business Finland.

### Competing interest statement

The authors declare no conflict of interest.

### Additional information

No additional information is available for this paper.
